# Ongoing guidance for infectious disease care for migrants and immigrants: a case-based narrative review

**DOI:** 10.1017/ash.2026.10390

**Published:** 2026-05-14

**Authors:** Rachel Croxton, Tessa Adžemović, Payal K. Patel, Elizabeth A. Scruggs-Wodkowski, Joseph Benigno Ladines-Lim

**Affiliations:** 1 Michigan Medicine, University of Michigan, Ann Arbor, MI, USA; 2 Mass General Brigham, Harvard University, Boston, MA, USA; 3 Intermountain Healthcare, Murray, UT, USA; 4 Penn Medicine, University of Pennsylvania, Philadelphia, PA, USA

## Abstract

In this narrative review, we build upon previous work and provide guidance on the infectious disease care of immigrant, refugee, and asylee populations, accounting for numerous policy changes at the federal level that have been made in the past year and with a focus on re-emerging infections such as measles. We present three cases to illustrate teaching points on the care of pertinent infections we have chosen (HIV, measles, and Ebola), not only at the patient level but also actions of advocacy that can be taken to build health systems that better care for these patients.

## Introduction

The passage of the H.R.1, also known as “One Big Beautiful Bill Act” (OBBBA), in the United States (US) on July 4, 2025, has resulted in cuts in funding to numerous governmental health agencies and programs, including the Centers for Disease Control and Prevention (CDC), Medicaid and Children’s Health Insurance Program (CHIP), and Affordable Care Act (ACA) subsidies, with an estimated increase of 1.3 million without health insurance in 2026.^1–5^ A White House executive order on January 20, 2025, and subsequent directives have also effectively dismantled the United States Agency for International Development (USAID),^6–9^ resulting in termination of over 80% of its programs.^10,11^ Additionally, the US has suspended refugee and asylum-seeker admissions,^12,13^ instructed Immigration and Customs Enforcement to remove individuals deemed not lawfully present in the country,^14,15^ and curbed the distribution of public services to any who physically arrive.^16^ Altogether, the new legislation and federal directives have been either found or projected to significantly impact health outcomes for vulnerable populations, both in the US^17–21^ and around the world.^22,23^

In prior work, we detailed guidance on caring for refugee and asylee populations, particularly those made vulnerable by human conflict, through three fictional but realistic cases covering multidrug-resistant infection, tuberculosis/HIV, and poliomyelitis.^24^ Here, we aim to expand upon that work by moving our focus from human conflict around the world to changes in US domestic and international policy as described above. As before, here we present three characteristic fictionalized cases, intended to provide practical guidance on adapting healthcare delivery to affected vulnerable communities.

## Case #1: HIV-positive newly arrived migrants and immigrants

A 12-year-old boy living with HIV presents to your clinic for routine care. He arrived in the US from South Sudan in December 2022 with his mother, who is also living with HIV, under protected refugee status. Both previously received their antiretroviral medications through a USAID-funded nonprofit agency. He is currently doing well and has an undetectable viral load on daily adherence to antiretroviral therapy (ART). He needs refills for this medication, but his mother has just learned that they no longer qualify for Medicaid due to the impact of OBBBA on affordable coverage for non-citizens.

### How do you proceed?

Children and adults living with HIV under protected refugee status currently have access to federal health programs such as Medicaid under the ACA.^25,26^ OBBBA will end availability of federally funded Medicaid and CHIP beginning on October 1, 2026.^4^ Only lawful permanent residents, Cuban/Haitian entrants, Compacts of Free Association migrants, and lawfully residing children and pregnant individuals under the CHIPRA 214 option will remain eligible for Medicaid, leaving many refugees and asylees without access to care. The Congressional Budget Office estimates that changes from OBBBA will result in 1.3 million individuals going without health care.^27^ For our patient and his mother, Medicaid eligibility will end when the bill goes into effect. Depending on the state where they reside, there may be state-funded programs for non-citizens to explore coverage, although notably large-scale federal cuts may put additional constraints on opportunities to expand or maintain these programs.^27^ Additionally, there may be free/low-cost clinic or charity-based health care options available in some regions, though these clinics may have limited ability and resources to expand services beyond what they currently provide.^28,29^

Alternative options for HIV medication and health care may remain available to our patient through the Ryan White HIV/AIDS Program (RWHAP) under the Health Resources & Services Administration. The RWHAP was established in 1990 to assist low-income people living with HIV to receive medical care, HIV treatment, and social services. It is roughly structured into 5 funding arms for: cities and counties most affected by HIV (part A); all 50 states to improve HIV medical and support services (part B); local community-based groups for outpatient ambulatory health services (part C); medical care for low-income women, infants, children, and youth living with HIV (part D); and AIDS education and training programs (part F).^30^ However, the FY 2026 Labor, Health and Human Services, Education, and Related Agencies bill advanced by the House Appropriations Committee has proposed $1.7 billion in cuts to federal HIV programs including $525.4 million in cuts (20% of the operating budget) for the RWHAP, which would eliminate parts C, D, and F.^31,32^ Budget cuts such as these, that reduce specialty medical care and support services for women, infants, children, and youth living with HIV, will further limit HIV care options for refugees and asylees given contraction of Medicaid services. If this proposed legislation is enacted, a substantial increase in HIV infections is predicted to occur.^33^

Clinicians caring for people living with HIV who may be affected by these legislative changes should make every effort to maintain uninterrupted ART when ART is indicated. Interruption of ART is not recommended because it can lead to viral rebound, immune decompensation, and clinical progression, and treatment interruptions may also contribute to antiretroviral resistance.^34,35^ Potential approaches to maintaining access may include short-term bridge prescriptions when feasible, evaluation for enrollment in Ryan White HIV/AIDS Program Part B AIDS Drug Assistance Program services, use of Ryan White or other 340B-covered clinic options, and referral to manufacturer patient assistance programs for eligible patients.^36,37^

## Case #2: Breakthrough case of measles infection

An 8-year-old girl from Guatemala presents to your emergency room with new high fever and malaise, as well as dry cough for the past several days. In the exam room, she is febrile with a temperature of 102.9°F, tachycardic with a resting heart rate of 130 beats per minute, and ill-appearing. She has rhinorrhea, injected conjunctiva, and tiny white lesions on the buccal mucosa. Other kids in her classroom have been sick recently as well. Her parents do not speak English well, but through a translator you learn that they have been living in the US for two years. Her vaccination status is unknown.

### How do you proceed?

In this case, there is a strong suspicion for measles infection, also known as rubeola. Infection control measures to prevent transmission, including immediate airborne isolation upon suspicion if hospitalization is required, are warranted.^38,39^ Guidance for providers may be offered from a health-system level. Diagnosis should be confirmed via laboratory testing, including measles IgM serology and real-time polymerase chain reaction (PCR) swab from either a nasopharyngeal/throat or urine specimen, with cases reported to local and state public health authorities.^38,39^ While supportive care is the mainstay of treatment, vitamin A supplementation has been found to reduce morbidity and mortality, with the recommended dosing varying by age.^40^ However, it is worth noting that vitamin A supplementation has not been well studied in the US,^41^ where vitamin A deficiency is rare. In fact, there have been reports of vitamin A toxicity associated with measles outbreaks, most recently in West Texas,^42,43^ highlighting the need to ensure communication of accurate medical guidance.^40^ Any exposed contacts, such as her parents, other schoolchildren, and potentially others, should receive postexposure prophylaxis if they lack confirmed immunity, which would be either measles-mumps-rubella vaccine within 72 hours or immune globulin within 6 days.^39,40^ The measles resurgence has affected over 40 states in 2025, which has been attributed to decreasing immunization rates.^44^ Federal actions have greatly altered the CDC, including its Advisory Committee on Immunization Practices, which has led to recommendations for significant changes in the routine childhood vaccine schedule^45^ and several professional societies and initiatives—i.e., the American College of Physicians, American Academy of Pediatrics, American College of Obstetricians and Gynecologists, American Academy of Family Physicians, and Vaccine Integrity Project—to release their own vaccine guidance.^46–51^

## Case #3: Ebola

A 32-year-old Ugandan home health aide recently returned from a trip to Uganda. She came back seven days ago and was in her usual state of health up until yesterday, when she developed a high fever, myalgias, nausea, abdominal pain, and profuse diarrhea. On exam, she is febrile with a temperature of 103.7°F, tachycardic with a heart rate of 120 beats per minute, and tachypneic with a respiratory rate of 22 breaths per minute. She has a diffuse rash and bleeding from her gums. While she was in Uganda, she was working at a rural community health site.

### How do you proceed?

This patient’s presentation raises suspicion for Ebola virus disease (EVD), a highly contagious hemorrhagic febrile illness.^52^ The typical incubation period is 2–21 days. As a healthcare worker, she is at increased risk of being exposed and exposing others, particularly immunocompromised patients. It is imperative that she be isolated immediately and contact tracing be performed. It is important that appropriate Personal Protective Equipment be used, in accordance with CDC viral hemorrhage protocol.^53^ The clinician will need to contact their hospital infection prevention team immediately. Public health officials can assist and should be contacted as soon as possible, including the state health department and CDC Emergency Operations Center. She is at risk of multiorgan failure, shock, and death. Diagnosis is made via whole blood quantitative reverse transcription-PCR assay. There are multiple identified strains of EVD, including Zaire ebolavirus and Sudan ebolavirus, with most recent outbreaks of EVD due to the latter. EVD has a high fatality rate, though it is substantially lower in the US than in Africa.^54^ Treatment for EVD includes supportive care (eg, intravenous fluid resuscitation, oxygen), and for Zaire ebolavirus, there are US Food and Drug Administration-approved monoclonal antibodies, specifically Inmazeb® (atoltivimab/maftivimab/odesivimab) and Ebanga™ (ansuvimab).^52^

In the United States, there is a tiered approach to care of the patient with Ebola Virus. Patients are first assessed at frontline facilities. If disease is suspected, they are taken to a designated assessment hospital. If the case is confirmed, the patient is transferred to an Ebola Treatment Center. This system was designed to prevent facility-level outbreaks of EVD.

The landscape of EVD care has changed greatly in the last ten years, with decreased fatality rates across the world and faster outbreak responses. However, with the dissolution of USAID and decreased funding to organizations like the World Health Organization, effective management of communicable, highly virulent diseases such as EVD is becoming more challenging. In February 2025, four of five USAID contracts dedicated to the Ebola response were terminated exactly at the time of the most recent outbreak,^55^ greatly hindering response efforts.

## Conclusion

Delivering care to refugee, asylee, and immigrant communities beset by human conflict around the world is a tremendous challenge for numerous reasons we have previously described.^24^ These communities, as well as native-born populations in the US, have been further impacted by domestic and international policy changes that have generally cut health care and other social services and placed severe restrictions on the refugee and immigrant population, which may have adverse effects on health outcomes for many years to come.^17–23^

**Figure 1.**
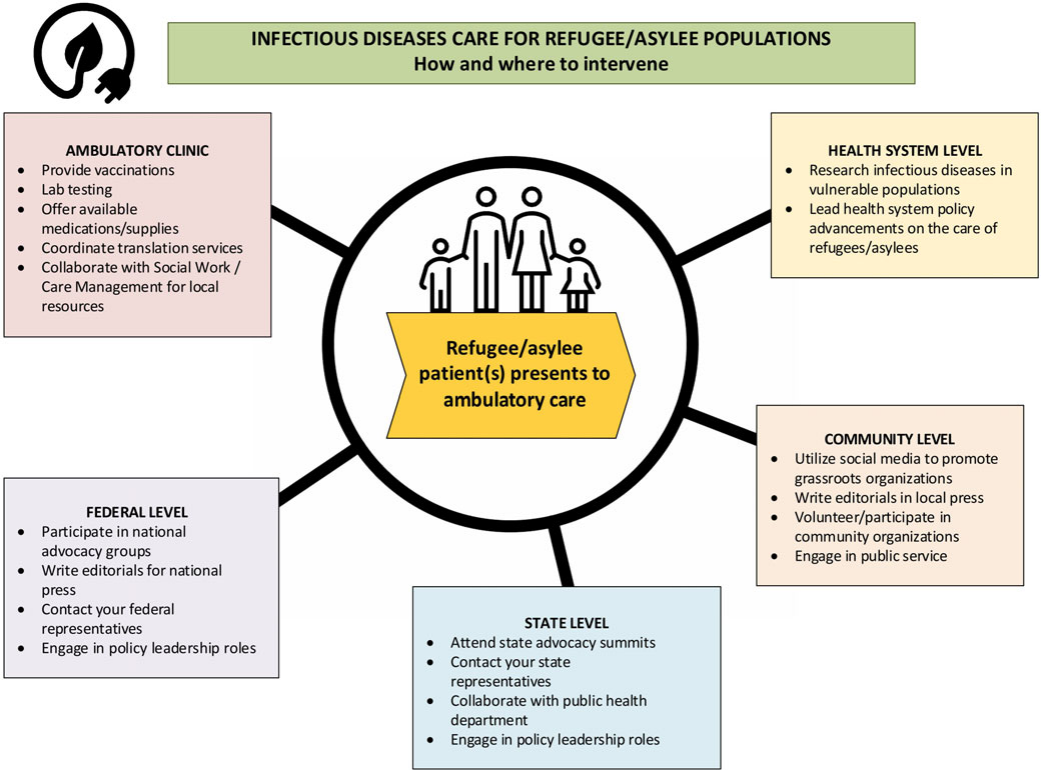
Recommendations for caring for refugee/asylee populations at the clinic, institutional, community, state, and federal levels.

We have presented three cases to illustrate the impact of policy changes on a hypothetical, individual level, including continuing care for HIV-positive individuals, treating vaccine-preventable infections that are resurging, such as measles, and addressing emerging infections such as Ebola that still may affect those living in the US. While not discussed in the individual cases outlined above, we also recommend that Infectious Diseases clinicians should consider how to intervene beyond the patient level to address the impact of policy changes on (re-)emerging infections, antimicrobial resistance, and the management and prevention of HIV more broadly. We echo the call to seek opportunities at the community, state, and national level to advocate for vulnerable populations who may disproportionately be impacted by cuts in healthcare funding and research in infectious diseases.^56^ We have outlined some potential places to intervene in Figure 1, which shows our own original graphic.

We note that this review is not intended to serve as a complete replacement or substitute for the resources that address the topics in our fictitious cases, which we have referenced. Still, we continue to experience the effects of the policy changes of the current administration, and in an ever-evolving landscape of emerging and re-emerging infectious diseases, we hope this work serves as practical guidance for clinicians caring for vulnerable populations, both native- and foreign-born, in the US.
